# Inequities in PrEP annualized pill‐day coverage, United States, 2018–2022: a cross‐sectional pharmacoequity analysis

**DOI:** 10.1002/jia2.26459

**Published:** 2025-05-13

**Authors:** Patrick S. Sullivan, Eric Hall, Heather Bradley, Elizabeth S. Russell, Cory R. Woodyatt

**Affiliations:** ^1^ Rollins School of Public Health Emory University Atlanta Georgia USA; ^2^ OHSU‐PSU School of Public Health, Oregon Health Sciences University Portland Oregon USA; ^3^ Merck & Co., Inc. Rahway New Jersey USA; ^4^ OHSU Department of Emergency Medicine Portland Oregon USA

**Keywords:** HIV, HIV prevention, PrEP, pre‐exposure prophylaxis, pharmacoequity, epidemiology

## Abstract

**Introduction:**

Pre‐exposure prophylaxis (PrEP) is highly effective in reducing the risk of HIV acquisition, but the population‐level impact of PrEP depends on the proportion of people with PrEP indications who use it (coverage) and how long they stay on it while at risk (persistence). We aimed to assess the extent to which PrEP persistence varied by race/ethnicity, sex and age.

**Methods:**

Previously reported methods and US commercial pharmacy data identified PrEP users and days covered. We calculated PrEP Days Covered (PDC) as the annual number of pills dispensed (i.e., pill‐days) overall and by sex, race/ethnicity and age group. Statistical differences by demographic characteristics were calculated. To assess the potential impact of 2‐1‐1 PrEP dosing on median days of PrEP use, we compared 2018 and 2022 (pre‐ and post‐US Public Health Service guideline for 2‐1‐1 dosing).

**Results:**

There were 225,180 PrEP users in 2018, and 459,984 in 2022. In 2022, the median PDC was 167 (IQR: 67, 308). There were 90 versus 180 median PDC for female and male users, respectively (difference of 90 PDC, 95% CI, 89.6−90.4). Among PrEP users with race/ethnicity data, the median PDC was higher for White non‐Hispanic (NH) (290 days) than Hispanic (268 days) or Black NH (251 days) users. Older users had significantly more PDC than younger users (<16 years: 60 days; 16–29 years: 120 days; 30–64 years: 191 days). Residents of states with PrEP‐Drug Assistance Programs (PrEP‐DAP) or Medicaid expansion had higher median PrEP duration than states without programmes. Median days covered for 2018 (154 days) and 2022 (167 days) did not suggest that the addition of the 2‐1‐1 PrEP guideline was associated with fewer covered days.

**Conclusions:**

PrEP programmes are often evaluated by enumerating people who used PrEP at any time during a year; our data indicate that significant differences in days of PrEP covered among users might mask further inequities in PrEP protection among women, and Black, Hispanic and younger people. Evaluations of PrEP equity should include a pharmacoequity component by assessing days covered as an additional indicator of PrEP equity.

## 1 INTRODUCTION

Fourteen years after the first efficacy evidence for pre‐exposure prophylaxis (PrEP) was published [1], daily oral PrEP is now understood to be almost 100% effective in adherent individuals. Despite this, the uptake of PrEP among people with PrEP indications in the United States has been slow [2, 3], and as of 2024, only about one‐third of people with PrEP indications were taking the medication [4]. Further, the effectiveness of PrEP as evaluated by real‐world data; barriers to adherence include health concerns, side effects and concerns about the adequacy of PrEP protection [5]. Therefore, data are needed to understand persistence, and persistence should be monitored on a population basis to understand the impact of PrEP programmes.

In addition to opportunities to reduce new HIV acquisitions through increasing the number of people receiving PrEP, we must also prioritize getting PrEP to the people who are most likely to benefit from it—PrEP equity [6]. Equitable PrEP use has been proposed to be monitored by the PrEP‐to‐Need ratio (PnR) [7], an equity metric that standardizes levels of PrEP use in certain groups (e.g., women, men, Black, Hispanic or younger people) to the epidemic burden in that group.

Assessments of PrEP equity through the first decade of PrEP availability in the United States have indicated that women, Black people, Hispanic people, younger people and people in the US South have lower levels of PrEP use relative to their epidemic needs—and that inequities in use of PrEP have grown during the first decade of its use [8]. Inequities in PrEP by race/ethnicity are largely related to structural factors, including geographic proximity to PrEP services [9, 10], stigma experienced or anticipated in PrEP services and other medical settings [6, 11], and missed policy opportunities in Southern US states [12], which have high populations of Black residents.

Monitoring of PrEP at the population level and PrEP equity have relied on commercial prescription data [13]. These data have important limitations, including limitations in the availability of data on race and ethnicity [8]. Further, the primary metric utilized in public health monitoring has been PrEP pill coverage [2, 8, 13]. For many analyses, PrEP use in a year has been defined as having at least 1 day of PrEP prescription during that year [2, 3, 14], with the notable exception of the work of Huang et al. [13]. Such assumptions are simplistic and become more problematic to estimating PrEP coverage as more flexible PrEP oral regimens (e.g. 2‐1‐1 PrEP) [15, 16] become more commonly used. The availability of long‐acting injectable (LAI) PrEP, which has shown higher efficacy for HIV prevention than daily oral PrEP for both men who have sex with men (MSM) and women [17, 18, 19], will also require the development and evaluation of new methods for monitoring PrEP use. These might require additional studies to develop and test methods to monitor new PrEP regimens (e.g. 2‐1‐1) through commercial pharmacy data, the addition of questions to ongoing public health surveillance systems [20] and new analyses of administrative data from PrEP Drug Assistance Programs (PrEP‐DAP).

We aimed to move beyond previous analyses addressing any PrEP use in a year to consider pharmacoequity—the extent to which the patterns of PrEP use are equitable in terms of the differential epidemic risk among different subgroups of people in the United States. Here, we considered the duration of PrEP use (as opposed to a dichotomous used PrEP/did not use PrEP in a year) and evaluated duration by age, sex and geographic region. We believe that the duration of oral PrEP use is an additional important indicator in identifying nested inequities in effective PrEP protection.

## 2 METHODS

### 2.1 PrEP prescriptions

We used commercial pharmacy data (IQVIA, Durham, NC) to identify PrEP prescriptions, using methods previously reported [3]. These data include information about prescriptions dispensed by more than 90% of retail pharmacies and use national estimates of prescription fills to estimate PrEP prescriptions for the small percentage of US prescriptions that are not tracked directly by IQVIA. Federal and State PrEP programmes are included, to the extent that prescriptions from them are filled through pharmacies. Because the data do not include identifiers, human subjects review was not required. All analyses were conducted in SAS.

We used a previously developed algorithm [3] to differentiate medication use for HIV treatment from use for HIV PrEP by identifying mono‐therapy patients. First, we identified all patients who were on Emtricitabine‐Tenofovir Disoproxil Fumarate (branded and generic Truvada), Emtricitabine‐Tenofovir Alafenamide Fumarate (Descovy) and/or Cabotegravir (Apretude) during the analysis period. To exclude uses of these agents for treatment, we looked back to previous months of data and removed patients who had been on any antiretroviral treatment other than the three target medications at any point during the analysis period [21]. IQVIA data do not identify individuals but do include data on sex (but not gender), state and county of US residence, and age. A subset of records also has data on race and ethnicity, which are determined by IQVIA using probabilistic matching of prescription data with data from consumer credit reporting systems.

### 2.2 Duration of PrEP use

Using commercial pharmacy data, we calculated the duration of PrEP use by using dates of all prescriptions in a year and number of pills dispensed (i.e., pill‐days per year). PrEP users include all patients who, at any point during the year filled a prescription for Emtricitabine‐Tenofovir Disoproxil Fumarate, Emtricitabine‐Tenofovir Alafenamide Fumarate and/or Cabotegravir, but were not on any other antiretroviral medications.

### 2.3 Median length of PrEP use

Median length (and interquartile range) of PrEP use was calculated for all US users by year from 2018 to 2022 overall, by sex, by race/ethnicity and by age groups. We chose to present median, rather than mean, values because the data for PrEP pill days were right‐skewed. We believed that the median was a more appropriate measure for this research question, as the comparisons were less likely to be influenced by extreme observations towards very low or high pill days (see Supplementary Table for data on means). We fit quantile regression models to estimate the difference in median days on PrEP per year (and corresponding 95% confidence intervals) by sex, race/ethnicity and age groups. Race/ethnicity was missing for 75% of records, so the average of pill days in stratified race/ethnicity data are different than the average among all people in the overall sample.

### 2.4 2‐1‐1‐Dosing

To assess the impact of 2‐1‐1 PrEP dosing on median days of PrEP use, we compared data from 2018 and 2022 (pre‐ and post‐US Public Health Service [USPHS] guideline for 2‐1‐1 dosing) [22]. Our hypothesis was that increased use of 2‐1‐1 PrEP at a population level could be recognized by lower pill‐day coverage in the 2022 data, when 2‐1‐1 PrEP was included in clinical guidelines. Medians among groups were compared with median tests, with an alpha of 0.05. We assessed for differences by sex, age, race/ethnicity and US Census region.

Ethics approval was not obtained as no personally identifying information was used in the analysis. The data sets obtained from IQVIA did not contain personally identifying information.

## 3 RESULTS

**Figure 1:**
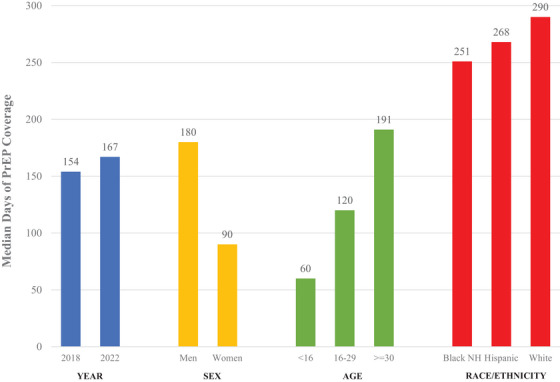
Median days of pre‐exposure prophylaxis (PrEP) coverage by year, sex, age and race ethnicity, 2018 and 2022, United States. NH, non‐Hispanic.

We evaluated data from 225,180 PrEP users in 2018 and 459,984 PrEP users in 2022. 2016 and 2017 are not complete because to be eligible for this data pull, there had to be at least one visit in 2018 or later. The distribution of all 2022 PrEP prescriptions (according to our definition) by marketed name is provided in Table 1. In 2022, the median number of days covered by a PrEP prescription among PrEP users was 167 (Table 2). Median days were lower among female (90 days) than male (180 days) users. Among PrEP users, median days of use were significantly higher for White non‐Hispanic (290 days) than for Hispanic (268 days) or Black (251 days) PrEP users. Older users had significantly higher numbers of days covered by PrEP than younger users <16 years: 60 days; 16–29 years: 120 days; 30–64 years: 191 days; 65 and older: 180 days (Figure 1). Note that all data are for 2022, except the 2018 bar in the year grouping.

**Table 1:** Distribution of 2022 pre‐exposure prophylaxis (PrEP) prescriptions by marketed name in the United States

Marketed product name	*n*	%
Apretude	11,928	0.50
Descovy	1,107,655	46.42
Emtricitabine/tenofovir D	1,170,043	49.04
Truvada	96,298	4.04
	2,385,924	

**Table 2:** Median days on PrEP by demographic characteristics of pre‐exposure prophylaxis (PrEP) users, United States, 2022

	Median	25th Percentile	75th Percentile	Difference	95% CI	
Overall	167	67	308			
Sex						
Female	90	30	180	Ref		
Male	180	77	314	90	89.6	90.4
Unknown	57	30	90			
Race/Ethnicity						
Asian	283	150	355	32	23.7	40.3
Black	251	116	343	Ref		
Hispanic	268	123	346	17	10.9	23.1
White	290	151	355	39	33.7	44.3
No match or unknown	128	60	270			
Age						
Missing	30	17.5	75			
<16 years	60	30	203	−60	−70.9	−49.1
16−29 years	120	60	256	Ref		
30−64 years	191	86	329	71	70.1	71.9
65+ years	180	70	338	60	58.5	61.5
Region						
Midwest	180	74	315	29	26.5	31.5
Northeast	170	64	309	19	16.7	21.3
South	159	64	302	8	5.5	10.5
West	151	62	297	Ref		
Missing	180	77	326			
Medicaid expansion						
Never	154	62	300	Ref		
Before 2018	165	65	305	11	8.4	13.6
After 2018	177	75	308	23	18.9	27.2
Missing	180	77	326			
PrEP‐DAP						
No	165	64	307	Ref		
Yes	161	65	302	−4	−6.2	−1.8
Missing	180	77	326			

Abbreviations: CI, confidence interval; PrEP‐DAP, Pre‐Exposure Prophylaxis Drug Assistance Program.

There were also significant differences in the median days on PrEP by US region: among those prescribed PrEP, users in the Midwest and Northeast had the highest median days on PrEP (180 and 170, respectively); those in the South (159 days) and West (151 days) had a lower median duration of PrEP. States with Medicaid expansion, before or after 2018, had a higher duration of PrEP use compared to states that have not yet expanded Medicaid (Table 2). States with PrEP‐DAP programmes had a slightly lower, but significantly different median duration of coverage than did states without PrEP‐DAP programmes.

A comparison of overall data for 2018 (154 days) versus 2022 (167 days) did not suggest that the USPHS 2021 inclusion of 2‐1‐1 PrEP in PrEP guidelines was associated with a reduction in observed median days of coverage.

## 4 DISCUSSION

Data on the duration of PrEP use among US PrEP users suggest that the inequities in PrEP initiation by race [7] and age are likely compounded by inequities in the duration of PrEP protection. PrEP protection among people with PrEP indications is ultimately the conceptual product of the number of people with indications who receive and fill a PrEP prescription and the duration of their PrEP use (assuming risk is consistent over time). Prior analyses of population‐level PrEP data have documented that Black and Hispanic Americans, women, younger people and people who live in the US South are inequitably served by PrEP relative to their epidemic need [7, 8], using a metric of any PrEP prescription in a year [8]. According to our data, those same groups also experience shorter durations of PrEP use, compounding the deficits and inequities in PrEP protection associated with lower uptake of PrEP in these communities.

Neither PrEP equity by race nor stratified PrEP duration data has been described in the biomedical literature outside of the United States, although lower access to PrEP overall has been described in Africa for younger people and men [23]. The reasons that PrEP duration may be less in some groups compared to others is likely largely shaped by social determinants of health. Qualitative studies suggest that PrEP discontinuation among MSM is most shaped by the diminution of perceived risk [24], costs of medications [24], experienced or anticipated side effects and challenges with medication adherence [25]. Among women, PrEP persistence is challenged by limited access, cost, stigma and medical distrust [26]. Among young people, PrEP discontinuation has been reported to be associated with distance to care and female sex [27]. The diverse reasons for PrEP discontinuation, and continued risk after discontinuation [28], suggest that individualized discussions with PrEP users about barriers to PrEP persistence are needed. For example, preferences for PrEP options, including LAI PrEP, daily oral PrEP and event‐driven PrEP vary by age, characteristics of sex partners, race/ethnicity and region of residence [29, 30, 31, 32, 33, 34, 35].

The centrality of social drivers of PrEP discontinuation suggests that programmes will need to consider a package of steps to promote persistence on PrEP regimens. These facilitators of PrEP persistence might include programmes that pay for the costs of medications, the costs of wraparound services or both [12, 36]; PrEP formulations that require fewer trips to healthcare providers [37, 38]; PrEP options that minimize concerns about stigma associated with pill carrying [39]; PrEP delivery sites or services that minimize commute time and visit frequency [30]; as well as social support and self‐efficacy and adherence counselling [40, 41, 42].

The regional, age, sex and race/ethnicity differences in the duration of PrEP coverage are likely reflective of the differential impact of the mechanisms identified above. That certain groups (young people, Black and Hispanic people, people in the US South, women) experience both lower levels of PrEP prescription relative to their epidemic burden [8] and shorter duration of PrEP prescription suggests that the differential lack of benefit from equitable PrEP prescriptions is reinforced by shorter duration of prescription among those who start PrEP. This is especially concerning because modelling data suggests that inequitable PrEP access is likely to result in expanding disparities in new HIV acquisitions for underserved groups [43].

Notably, the impacts of state decisions on Medicaid expansion and the availability of state PrEP‐DAP were associated with different directions of effect in terms of PrEP longevity. PrEP‐DAP programme states include California, Colorado, District of Columbia, Florida, Illinois, Indiana, Iowa, Massachusetts, New York, New Mexico, Ohio, Oklahoma, Virginia and Washington. In states with Medicaid expansion, the median duration of PrEP prescriptions was longer. This is consistent with data suggesting that [3, 7, 12] PnR is higher in states that have Medicaid expansion [12]. Notably, the provision of PrEP‐DAP programmes at the state level was not associated with a longer duration of PrEP prescriptions; a prior report of PrEP use documented that states with PrEP‐DAP programmes as of 2018 experienced higher PnRs than states without such programmes [7]. This suggests that PrEP‐DAP programmes may be impactful in supporting more equitable PrEP starts, but not in mitigating the discontinuation of PrEP once prescribed.

The population‐level protection against HIV acquisition within populations will be determined by several factors: the extent to which those at highest risk for HIV are more likely to receive it (i.e. PrEP equity); the absolute proportion of coverage; the persistence on therapy once started; and provider's comfort and willingness to prescribe PrEP. Before this analysis, there were population‐level data to support inequitably low PrEP use by non‐White people, Hispanic people, women and people who live in the South [8]. Our findings document that disparities in PrEP persistence likely compound the gaps in initial prescriptions, leading to deficits in pill‐day protection for women, Black and Hispanic people, PrEP users aged <16 years and people who live in states without Medicaid expansion. Provider comfort with prescribing PrEP and provider knowledge and comfort with alternate PrEP dosing patterns will also shape the population level of protection. Not all providers are comfortable prescribing PrEP [44], and the amount of time required to counsel and prescribe PrEP is an important provider concern [45]. Exploring options for alternative dosing regimens for 2‐1‐1 PrEP might also increase the time required in clinical encounters.

PrEP is highly effective at reducing HIV transmissions [46], but social drivers of access to PrEP also extend to PrEP persistence. Based on prior studies around the barriers to PrEP uptake and equitable PrEP use, it seems likely that interventions to increase proximity to PrEP providers, providing PrEP choices and addressing concerns about out‐of‐pocket costs for PrEP care might be important steps to address gaps in PrEP retention. Clients for whom transportation concerns [47], privacy concerns [39] or daily pill‐taking challenges limit PrEP duration could benefit from alternative PrEP formulations, including LAI PrEP [38]. Additional research is needed to understand the preferences for LAI PrEP in the groups most impacted by gaps in PrEP prescription and persistence [48].

There are several important limitations to our analysis. First, commercial PrEP data do not include all sources of PrEP fulfillment, so our analyses are based on a subset of PrEP users. It is not known whether PrEP users not included in our commercial pharmacy data might have different patterns of use compared to those included in our analysis. Second, misclassification of medication as PrEP was possible in that there might be differences in the performance of the algorithm used to identify PrEP prescriptions for different PrEP medications and dosing schedules [21]. There is a risk that a very small proportion of people identified as using Descovy for PrEP could have been living with HIV. Third, data about the characteristics of PrEP users (age, race/ethnicity, sex) might be subject to misclassification and race/ethnicity data have high levels of missingness; even the limited data on race/ethnicity available might not be representative of the IQVIA sample or the population. This is an important limitation because of the profound disparities in the burden of HIV acquisition by race/ethnicity in the United States. It is unknown whether the missingness of data is differential by some third factor associated with PrEP use, and therefore, further evaluations of the extent to which missingness of race varies by insurance, income or region of the United States will be important to understand and quantify any potential biases. Fourth, the commercial PrEP data exclude some practices that provide in‐house pharmacy services, and it is not known whether the PrEP users who have their prescriptions filled in these practices might have different patterns of PrEP use compared to users in the commercial pharmacy data. Fifth, our data does not account for intermittent PrEP use [15], which could lead to intermittent PrEP users being considered as people who discontinued PrEP. However, because data from only 2018 and 2022 were analysed, there is still a risk that a very small proportion of participants were HIV positive, particularly on Descovy. Lastly, it is possible that multiple changes in patterns of PrEP use occurred between the two time points that operated in opposite directions and obscured one another; for example, a higher proportion of people using 2‐1‐1 PrEP and higher adherence in people using daily oral PrEP might result in the lack of an apparent change in median days of PrEP, while underlying patterns of adherence were actually improving. Resolving such nuanced combinations of changes would require additional elements of data, such as dosing instructions, from commercial pharmacy datasets.

Duration of PrEP coverage should be considered as a monitoring element in national data collection systems to understand patterns of PrEP adoption and to help tailor PrEP choices to people who might benefit from PrEP. Future research efforts in this area might include adding explicit questions on alternative PrEP regimens to national surveys of MSM (e.g. National HIV Behavioral Surveillance surveys [20], American Men's Internet Survey [2]).

## 5 CONCLUSIONS

The population impact of PrEP will depend on the coverage of PrEP among those with indications [49], the equity of coverage in relation to risk in specific populations [8, 43] and persistence on PrEP [50]. PrEP coverage has been below a third since PrEP became available for use [51, 52], and there are substantial gaps in PrEP equity by race, sex and US region [8]. Greater attention is needed to PrEP persistence; understanding how to extend the persistence on PrEP once people start it is key to increasing the population impact of PrEP. We recommend monitoring PrEP persistence using population‐based data sources, smaller studies to understand the multilevel influences on PrEP persistence and intervention research to develop evidence‐based approaches to improve PrEP persistence. This future work should explicitly examine the associations within subgroups across social determinants of health, including race/ethnicity, region of residence, age and socio‐economic position with PrEP duration. Alternative methods of providing PrEP should be considered to improve PrEP persistence, especially for groups with documented shorter durations of PrEP persistence. LAI formulations might be especially impactful in increasing the duration of PrEP coverage for populations at the highest risk of experiencing PrEP or HIV stigma.

## COMPETING INTERESTS

The authors do not have competing interests to report.

## AUTHORS’ CONTRIBUTIONS

PSS conceptualized the analysis, interpreted the results and participated in the initial drafting and finalization of the manuscript, and approved the final manuscript. EH conceptualized the analysis, wrote code for statistical analyses, participated in the initial drafting and finalization of the manuscript, and approved the final manuscript. HB conceptualized the analysis, interpreted the results, participated in the finalization of the manuscript and approved the final manuscript; ESR conceptualized the analysis, interpreted the results, participated in the initial drafting and finalization of the manuscript, and approved the final manuscript; CRW provided administrative management, interpreted the results, participated in the initial drafting and finalization of the manuscript, and approved the final manuscript.

## FUNDING

This research was supported by Merck Sharp & Dohme LLC, a subsidiary of Merck & Co., Inc., Rahway, NJ, USA, and the Center for AIDS Research at Emory University (P30AI050409).

## Supporting information



Supporting Information

## Data Availability

Data on duration of use were developed from a proprietary database of pharmacy fills purchased from IQVIA. SAS code to analyse the data is available upon request; the underlying data are available from IQVIA,5 subject to their contractual processes.
